# *Trapa bispinosa* Roxb.: A Review on Nutritional and Pharmacological Aspects

**DOI:** 10.1155/2014/959830

**Published:** 2014-02-10

**Authors:** Prafulla Adkar, Amita Dongare, Shirishkumar Ambavade, V. H. Bhaskar

**Affiliations:** ^1^Department of Pharmacology, JSPM's Jayawantrao Sawant College of Pharmacy and Research, Hadapsar, Pune,Maharashtra 411028, India; ^2^Post Graduates Department of Pharmacology and Toxicology, JSPM's Jayawantrao Sawant College of Pharmacy and Research, Handewadi Road, Hadapsar, Pune, Maharashtra 411028, India; ^3^Department of Pharmaceutical Medicinal Chemistry, Gahlot Institute of Pharmacy Plot No. 59, Sector No. 14, Koparkhairane, Navi Mumbai, Maharashtra 400709, India

## Abstract

*Trapa bispinosa* Roxb. which belongs to the family Trapaceae is a small herb well known for its medicinal properties and is widely used worldwide. *Trapa bispinosa* or *Trapa natans* is an important plant of Indian Ayurvedic system of medicine which is used in the problems of stomach, genitourinary system, liver, kidney, and spleen. It is bitter, astringent, stomachic, diuretic, febrifuge, and antiseptic. The whole plant is used in gonorrhea, menorrhagia, and other genital affections. It is useful in diarrhea, dysentery, ophthalmopathy, ulcers, and wounds. These are used in the validated conditions in pitta, burning sensation, dipsia, dyspepsia, hemorrhage, hemoptysis, diarrhea, dysentery, strangely, intermittent fever, leprosy, fatigue, inflammation, urethrorrhea, fractures, erysipelas, lumbago, pharyngitis, bronchitis and general debility, and suppressing stomach and heart burning. Maybe it is due to photochemical content of *Trapa bispinosa* having high quantity of minerals, ions, namely, Ca, K, Na, Zn, and vitamins; saponins, phenols, alkaloids, H-donation, flavonoids are reported in the plants. Nutritional and biochemical analyses of fruits of *Trapa bispinosa* in 100 g showed 22.30 and 71.55% carbohydrate, protein contents were 4.40% and 10.80%, a percentage of moisture, fiber, ash, and fat contents were 70.35 and 7.30, 2.05 and 6.35, 2.30 and 8.50, and 0.65 and 1.85, mineral contents of the seeds were 32 mg and 102.85 mg calcium, 1.4 and 3.8 mg Iron, and 121 and 325 mg phosphorus in 100 g, and seeds of *Trapa bispinosa* produced 115.52 and 354.85 Kcal of energy, in fresh and dry fruits, respectively. Chemical analysis of the fruit and fresh nuts having considerable water content citric acid and fresh fruit which substantiates its importance as dietary food also reported low crude lipid, and major mineral present with confirming good amount of minerals as an iron and manganese potassium were contained in the fruit. Crude fiber, total protein content of the water chestnut kernel, *Trapa bispinosa* are reported. In this paper, the recent reports on nutritional, phytochemical, and pharmacological aspects of *Trapa bispinosa* Roxb, as a medicinal and nutritional food, are reviewed.

## 1. Introduction

**Figure 1:**
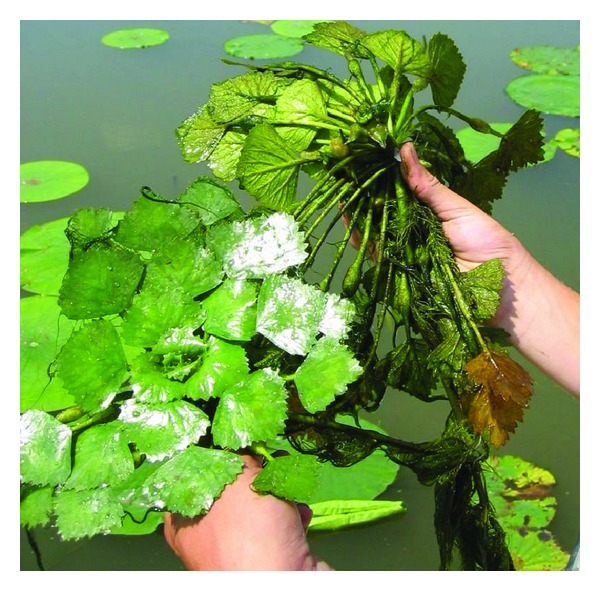
Whole plant *Trapa bispinosa*.

*Trapa bispinosa *Roxb(water chestnut) is an annual, floating-leaved aquatic plant (Figure 1) found in freshwater wetlands, lakes, ponds, and sluggish reaches of rivers in India [1, 2]. *Trapa bispinosa *is an aquatic floating herb which belongs to the family Trapaceae [3]. It has flexuose stem, ascending in the water; the submerged parts are furnished with numerous opposite pairs of green root-like spreading pectinate organs. Leaves are alternate, crowded on the upper part of the stem; 3.8–5 cm long, rhomboid, somewhat truncate at the base, irregularly inciso-serrate, reddish-purple beneath; petiole dilated near the apex. Flowers are few, auxiliary, solitary, pure white. Fruits obovoid, angular, 2.2–5 cm long, and broad, with a spreading flattened very sharp spinous horn at either side.

*Trapa bispinosa* is commonly grown throughout India and locally known as water chestnut [4]. In addition to being important for aquatic ecosystems, *Trapa bispinosa *species are also food for humans and animals in India, China, and Southeast Asia. It is grown throughout Asia and tropical Africa in lakes and ponds and is often cultivated for its edible fruit. The medicinal values of the whole herb and fruit have long been recognized in folklore medicine as a cure for various diseases [5].

*Trapa bispinosa *is an annual aquatic plant found in tropical and subtropical and temperate zones of the world. Their natural range of growth includes Southern Europe, Africa and Asia. It has been grown in Europe since Neolithic times. It is commonly used as food by ancient Europeans as an easy growing plant; it has become neutralized in part of USA since it was first introduced into North America in 1874. It was found in slow moving rivers, ponds, lakes, and damps and is widely cultivated in Asia. It favors nutrient rich water with pH range between 6.7 and 8.2 and the alkalinity between 12 and 128 mg/L of calcium carbonate [6].

## 2. Historical Perspectives

*Trapa bispinosa *had been introduced from Europe as an ornamental plant. Dispersal is limited because of the large, sinking nuts, but water chestnut has persisted and spread in the Northeastern states. In the Chinese Zhou Dynasty, water caltrop was an important food for worship as prayer offerings. The rites of Zhou (2nd century BC) mentioned that a worshipper should use a bamboo basket containing dried water caltrops. In India it is known as singhara or paniphal (eastern India) and is widely cultivated in fresh water lakes. The fruits are eaten raw or boiled. When the fruit has been dried, it is ground to a flour called singhare ka atta which is used in many religious rituals and can be consumed as a *Phalahar* diet on the Hindu fasting days, in Indian traditional festival “*Navratri*” [7].

The *Trapa bispinosa *is native to Eurasia. It was first introduced to North America in the 1870s, where it is known to have been grown in a botanical garden at Harvard University in 1877. The plant had escaped cultivation and was found growing in the Charles River by 1879.

## 3. Habitat

*Trapa bispinosa *Roxb. (Family: Trapaceae) is native to India. The fruit is commonly known as “Paniphal.” It grows abundantly in the lakes of Kashmir, India. The plant is commercially cultivated in tropical parts of the world such as Pakistan, Sri Lanka, Ceylon, Indonesia, and Africa. The plant is also abundant in Indonesia, southeast Asia, and the Southern part of China and in the eutrophic waters of Japan, Italy, and tropical America. It has become naturalized in a few places in the Eastern United States [9]. It is commercially cultivated across different parts of India for its consumable seasonal fruit commonly known as singhara which is a good source of nutrition having considerable amount of carbohydrate, protein, and vitamins. *Trapa bispinosa *Roxb plant floats just beneath the water surface and thus forms a thick mat in the water column. Only its upper leaves float over water surface in an artistic radial pattern with swollen, air-filled petioles that keep the upper part of the plant afloat [9].

*Trapa bispinosa *Roxb was first observed in North America, growing “luxuriantly” in Sanders Lake, Schenectady, New York, in 1884. The plant subsequently spread to many other areas in the Northeastern United States including Connecticut, Delaware, Maryland, Massachusetts, New Hampshire, Pennsylvania, Vermont, Virginia, and Washington D.C. The plant is now present in the Great Lakes Basin and recently has been found in Quebec, Canada.

## 4. Cultivation and Collection

*Trapa bispinosa* seedlings are transplanted in May/June in a perennial pond. These plants make use of the available organic matter for their growth. Stock of 800 (50 g) common carp fingerlings is maintained in September-October. *Trapa bispinosa* fruits ripen in winter and are harvested from November to January [4].

## 5. Botanical Description

See Figure 2.

## 6. Pharmacognostic Characters

*Trapa bispinosa* contains a great quantity of nonnutritional antioxidants, such as flavonoids, flavones, and total phenol contents. Flavonoids are present in plant tissues, such as fruits, vegetables, nuts, seeds, and leaves, in relatively high concentrations. Flavonoids act as natural antioxidants. Phytochemical screening of seed extract of *Trapa bispinosa* fruits reveals the presence of carbohydrates, saponins, phytosterols, fixed oils, and fat, while the pericarp extract of the fruits of *Trapa bispinosa* revealed the presence of tannins, flavonoids and glycosides alkaloids, saponins, steroids, and phenolic compound (Table 3) [10]. The literature reveals the presence of saponins, tannins, flavonoids, and glycosides in the pericarp extract of fruit [6]. The kernel is delicious and contains carbohydrates, proteins, and essential minerals. It also contains plentiful B vitamins (including B1, B2, B5, and B6), E, A, and C vitamins. Seeds also contain thiamine [4].

### 6.1. Growth Response

Dry weight per plant, percent cover, rosette number and rosette diameter of *Trapa bispinosa *increased significantly in all the five treatments during the experiment (*P* < 0.01 in all cases). At the first harvest before removal, the maximum value of dry weight per plant was obtained in the T9 treatment which differed markedly from the other four treatments (*P* = 0.006). Furthermore, dry weights per plant of *Trapa bispinosa *in the monocultures (T7, T8, and T9) were always higher than those in mixed-species treatments (T5 and T6). At the final harvest, the removal of *N. peltata* had a positive impact on dry weights of *Trapa bispinosa*. Mean dry weight per plant of *Trapa bispinosa *individuals was much higher in the *N. peltata* removed aquaria (the T5 treatment) than those in the control aquaria (the T6 treatment). However, the removal of *Trapa bispinosa *did not facilitate the growth of remaining individual plants of *Trapa bispinosa*. There were no significant differences in dry weight of *Trapa bispinosa *between the T7 and the T8. Similarly, at the 12th week, a clear difference was observed in percent cover among the +ve treatments (*P* < 0.001). In the T5 treatment, the cover of *T. bispinosa* reached 62% at the 12th week which was significantly high [10].

### 6.2. Photosynthetic Response and Carboxylation Activity

*Trapa bispinosa *plant often develops a large scale root system in natural waters. The roots are green colored with chlorophyll and capable of photosynthesis using light energy coming through the water. The production of this plant depends solely on the photosynthesis of these roots until leaves expand on and above the water surface to function in photosynthesis. Interestingly, photosynthesis in this plant is carried out fewer than two different environments, by roots in water and by leaves in the atmosphere. The researchers investigated photosynthetic features of green roots in terms of 0, evolution response, and activity of photosynthetic enzymes. Leaf photosynthesis exhibited a C3 type trait, but in roots photosynthetic system was comparative to that of the submerged type (SUM) showing an increased activity in phosphoenolpyruvate carboxylase (PEP Case) and malate accumulation in root cells at night [11].

## 7. Phytochemistry

*Trapa bispinosa* (singhara) contains many organic and inorganic constituents which are mentioned below.

### 7.1. Inorganic Constituents (Table 2)

Acids, minerals, calcium, phosphorus, iron, copper, manganese, magnesium, sodium and potassium [12], and the physico-chemical characteristics of *Trapa bispinosa *are shown in Table 1.

Biochemical analyses of fruits of *Trapa bispinosa* in 100 g showed 22.30 and 71.55% carbohydrate in fresh and dry fruits, respectively. The protein contents were 4.40% and 10.80% in fresh and dry fruits, respectively. The percentage of moisture, fiber, ash, and fat contents was 70.35 and 7.30, 2.05 and 6.35, 2.30 and 8.50, and 0.65 and 1.85, in fresh and dry fruits, respectively. The mineral contents of the seeds were 32 mg and 102.85 mg calcium, 1.4 and 3.8 mg iron and 121 and 325 mg phosphorus in 100 g, in fresh and dry fruits, respectively (Table 1). In 100 g fresh and dried seeds of *Trapa bispinosa *produced 115.52 and 354.85 Kcal of energy, respectively [13].

### 7.2. Chemical Composition of Water Chestnut Kernel

Chemical analysis of the fruit showed that the moisture content of *Trapa bispinosa *kernel was 81.12% (wet basis). Fresh nuts having considerable water content are taken at breakfasts and are believed to suppress stomach and heart burning. The total soluble solids content of the fruit was 7.2%. The total acid in terms of citric acid present was 0.142%. Negligible amount of fat content was noticed in the fruit as 0.36% which substantiates its importance as dietary food. Also reported low crude lipid content in Chinese water chestnut was 0.06%. Total ash content obtained in fruit was 1.33% confirming good amount of minerals contained in the fruit. The potassium content of 0.41% has been reported as the major mineral present with iron and manganese contents which were 0.21 and 0.08%, respectively, being the minor minerals present [12]. Crude fiber content of the water chestnut kernel was found to be 0.72% slightly higher than reported in Chinese *Trapa bispinosa* [12] as 0.60%. The total protein content calculated in the fruit was 1.87%. Low protein content has been earlier reported in *Trapa bispinosa.*

Previously isolated classes of constituents, ascorbic acid, amylase, and amylopectin were isolated from the fruits of *Trapa bispinosa *[14].

### 7.3. Organic Constituents

It contains carbohydrates and vitamins, namely, Vitamin B-complex (thiamine, riboflavin, pantothenic acid, pyridoxine, nicotinic acid), vitamin-C, vitamin-A, D-amylase, amylase, and considerable amount of phosphorylase [12]. Cycloeucalenol, ursolic acid, and 2*β*,3*α*,23-trihydroxyurs-12-en-28-oic acid [29].

The phytochemical content of *Trapa bispinosa *showed high quantity of saponins (36.92 ± 0.67%). Alkaloids present in the plants function as spasmolytic, anticholinergic, and anesthetic agents. The alkaloid content in *Trapa bispinosa* was found to be 0.775 ± 0.33%. Reports suggest that phenols antioxidant activity is due to their redox properties, H-donation, prevention of chain initiation by donating electrons or by binding transition metal ion catalysts, and singlet oxygen quenchers. Flavonoids are important for their pharmacological activities as scavengers. Flavonoids prevent platelet stickiness and hence platelet aggregation. Colorimetric study of the two extracts of *Trapa bispinosa *showed that acetone solvent system was able to extract more phytochemicals in comparison to DCM : Me OH.

### 7.4. Chemical Constituents

See Figure 3.

## 8. Ethnopharmacology

*Actions* are aphrodisiac, astringent, appetizer, anti-pyretic, constipating, diuretic, haemostatic, refrigerant, nutritive, anti-diarrheal, and tonic [30].

*Indications* are dyspepsia, diarrhea, dysentery, strangury, intermittent fevers, leprosy, pharyngitis, lumbago, bronchitis, sore throat, hemorrhage, generalized debility, leucorrhea, threatened abortion, dysuria, and inflammation [12, 30]. The fruits are used as intestinal astringent, aphrodisiac, and antiinflammatory, and in leprosy, urinary discharges, fractures, sore throat, and anemia [14].

In Kashmir the water nuts form a staple farinaceous food. Fruit or nut or seed contains manganese and starch. It is nutritive, sweet, tonic, and cooling. Fresh fruits are edible, raw, and cooked; dried ones are baked and eaten. They are also grated into flour and made into cakes. The nutritive value of the kernels is shown by analysis to be equal to that of rice. Fruits are refrigerant and useful in diarrhea and bilious affections with diarrhea. The upper portion of the stem was used in poultices as a discutient and the expressed juice in eye diseases [31].

### 8.1. Uses in Unani Medicine

It is used in cases of sexual debility, spermatorrhea, general debility, fatigue, tuberculosis, intermittent fevers, dysentery, dry cough bilious affections [32], bleeding disorders, anal fissure, lumbago, dental caries [32], and sore throat.

### 8.2. Uses during Pregnancy, Sexual Transmitted Disease and Fertility

If there is itching on her lower abdomen, thigh or, breast, take water chestnut (*Trapa bispinosa*) lotus [31].

Water chestnut fruits with milk are used in nervous and general debility, seminal weakness and leucorrhoea, as confection made of it is given in 2 to 4 doctor doses. In menorrhagia hakims prescribe it as a compound powder thus; Take of *Trapa bispinosa*, kamarkas (kino) and white sugar. Divide them into 7 parts and take 1 part every day [31].

## 9. Nutritional Aspects

Biochemical composition of fruits of *Trapa bispinosa *was studied and concluded that *Trapa bispinosa* could be important sources of carbohydrate, protein, and minerals, which is suitable for incorporation in human diet [13].

Nutrient composition of water chestnuts revealed moisture 62.5, ash 1.04, crude fiber 2.13%, total soluble sugar 0.92%, reducing sugar 0.33%, nonreducing sugar 0.59%, starch 8.7%, lipid 0.84%. One hundred gram of green variety contained water soluble protein 0.275 mg, beta-carotene 60 microg, vitamin-C 1.1 mg, and total phenol 0.5 mg. The minerals contents of green variety were potassium 5.22%, sodium 0.64%, calcium 0.25%, phosphorus 6.77%, sulphur 0.38%, and iron, copper, manganese, and zinc 200, 430, 90, and 600 ppm, respectively. The red variety contained moisture 62.7%, ash 1.30%, crude fiber 2.27%, total soluble sugar 0.90%, reducing sugar 0.30%, nonreducing sugar 0.60%, starch 8.2%, and lipid 0.83%. The red variety contained water soluble protein 0.251 mg, beta-carotene 92 microg, vitamin-C 0.9 mg, and total phenol 0.60 mg per 100 g. The red variety contained potassium 5.32%, sodium 0.59%, calcium 0.26% phosphorus 6.77%, sulphur 0.32%, iron 200 ppm, copper 450 ppm, manganese 110 ppm, and zinc 650 ppm. The free amino acids, glutamic acid, tryptophan, tyrosine, alanine, lysine, and leucine were commonly found in both varieties. In addition, green and red varieties contained cysteine, arginine and proline, and glutamine and asparagines, respectively. Thus, the present study sheds light on the nutrient contents of the two varieties of water chestnuts and suggests that water chestnuts may play a crucial role in human nutrition [33].

## 10. Pharmacology (Table 4)

### 10.1. Acute Oral Toxicity Study

Acute oral toxicity study of *Trapa bispinosa *was carried out in albino rats. The animals were divided into eight groups of six in each. The animals were fasted overnight prior to the acute experimental procedure. Karber's method was used to determine the dose; acacia gum (2% w/v) was used as vehicle to suspend the extracts and administered intraperitoneally. The control group received 2mL/kg of the vehicle intraperitoneally. The other group received the extract as test drug in one of the following doses: 100, 200, 400, 800, 1000, 2000, and 3000 mg/kg in a similar manner. Immediately after dosing, the animals were observed continuously for first four hours for behavioral changes and for mortality at the end of 24 hrs, and 48 hrs and 72 hrs, respectively. The toxicity study showed that the hydroethanol extract of drug at a minimum dose of 200 mg/kg onwards shows the reaction in experimental animals. However, no mortality was reported even after 72 hours. This indicates that the hydroethanol extract is safe up to a single dose of 3 g/kg body weight [17].

### 10.2. Analgesic Activity

Analgesic activity of the methanolic extract of the *T. bispinosa* root at a dose of 200 mg/kg and 400 mg/kg was evaluated against the standard drug pentazocine at a dose of 30 mg/kg. Adult Swiss albino mice of either sex of six numbers in each group were undertaken for study and evaluated by tail flick and tail immersion method.

Both doses of *T. bispinosa* roots methanolic extract were found to produce significant (*P* < 0.01) analgesic activity. In tail flick method, the extract at 200 mg/kg showed significant activity (*P* < 0.01) after 45 minutes, but in tail immersion method, the extract showed significant activity at all tested dose levels after 30-minute interval. The results showed significant analgesic activity against stimuli [17].

### 10.3. Antidiabetic Activity

To evaluate the antidiabetic activity of methanol extract of *T. natans* fruit peels (METN) in Wistar rats, the effect of METN on oral glucose tolerance and its effect on normoglycemic rats were studied. Diabetes was induced in rats by single intraperitonial injection of streptozotocin. Three days after STZ induction, the hyperglycemic rats were treated with METN orally at the dose of 100 and 200 mg/kg body weight daily for 15 days.

METN at the dose of 100 and 200 mg/kg orally significantly (*P* < 0.001) and dose dependently improved oral glucose tolerance exhibited hypoglycaemic effect in normal rats and antidiabetic activity in STZ-induced diabetic rats by reducing and normalizing the elevated fasting blood glucose levels as compared to those of STZ control group [34].

### 10.4. Antiulcer Activity

The antiulcer activity of the fruits of *Trapa bispinosa* was studied on Wistar rats. The antiulcer activity of 50% ethanolic extract at two dose levels was evaluated by using pyloric ligation and aspirin plus pyloric ligation models. The tests extract revealed significant antiulcer activity, which might be due to increase in total carbohydrate content and alter state of mucosal barrier of the stomach. The results indicate that the ethanolic extract of fruits of *Trapa bispinosa* is endowed with potential antiulcer activity [16].

### 10.5. Neuropharmacological Activity

The different doses (100, 250, 500 mg/kg, p.o) of hydroalcoholic extract of *Trapa bispinosa* were administered in laboratory animals. The effects of extract on various parameters, like motor coordination, spontaneous locomotor activity, object recognition, transfer latency, anxiolytic activity, and sodium nitrite induced respiratory arrest and hypoxic stress, and so forth, were studied. The *Trapa bispinosa* (250 & 500 mg/kg) was found to decrease time required to occupy the central platform (transfer latency) in the elevated plus maze and to increase discrimination index in the object recognition test, indicating nootropic activity. *Trapa bispinosa* (250 & 500 mg/kg) showed significant increase in reaction time in hot plate analgesic activity. Moreover, it also showed significant reduction in spontaneous locomotory activity and latency memory which may be due to enhanced cholinergic function. It also showed significant analgesic activity [19].

### 10.6. Nootropic Activity

*Trapa bispinosa *extract showed significant facilitatory effect and aversively investigated for its nootropic activity using various experimental paradigms of learning and memory, namely, transfer latency (TL) on elevated plus maze, passive avoidance response (PAS) and object recognition test. The investigation reported that *Trapa bispinosa* 500 mg/kg significantly reduced the TL on 2nd and 9th day while *Trapa bispinosa *250 mg/kg was found effective on 9th day. *Trapa bispinosa* 250 and 500 mg/kg significantly increased the step-down latency in the PAS at acquisition and retention test, 250 and 500 mg/kg motivated learning and memory in mice as well as improvement of memory in absence of cognitive deficit. From the above experiment it was proved that the hydroalcoholic extract of *Trapa bispinosa *had significant nootropic activity [7].

### 10.7. Neuroprotective Activity

Effect of hydroalcoholic extract of *Trapa bispinosa* was studied on fluorescence product and biochemical parameters like lipid peroxidation, catalase activity, and glutathione peroxidase activity in brain of female albino mice. Ageing was accelerated by the treatment of 0.5 mL 5%D-galactose for 15 days. This resulted in increased fluorescence product, increased lipid peroxidation and decreased antioxidant enzyme like glutathione peroxidase and catalase in cerebral cortex. After cotreatment with hydroalcoholic extract of *Trapa bispinosa* (500 mg/kg, p.o) there was decrease in fluorescence product in cerebral cortex. Moreover, *T. bispinosa* inhibited increased lipid peroxidation and restored glutathione peroxidase and catalase activity in cerebral cortex as compared to ageing accelerated control group [15].

### 10.8. Immunomodulatory Activity

In a study, the immunomodulatory potential of aqueous extract of fruits of *T. bispinosa* was scrutinized in experimental animals. The immunomodulatory effect was assessed in rats against sheep red blood cells as antigen by studying cell-mediated delayed type hypersensitivity reaction, humoral immunity response, and percent change in neutrophil count. Macrophage phagocytosis assay was carried out by carbon clearance method in mice. Oral administration of TBAE dose dependently increased immunostimulatory response. Delayed type hypersensitivity reaction was found to be augmented significantly (*P* < 0.05) by increasing the mean foot pad thickness at 48 hr and production of circulatory antibody titer (humoral antibody response) was significantly (*P* < 0.05) increased in response to SRBC as an antigen. In addition, immune stimulation was counteracted by upregulating macrophage phagocytosis in response to carbon particles. Immunostimulatory property of TBAE further confirmed by elevated neutrophil counts was significantly (*P* < 0.01) compared to control values. The result of this study suggests that aqueous extract of fruits of *T. bispinosa* could stimulate the cellular and humoural response in animals [15].

### 10.9. Antifungal and Antimicrobial Activity

In recent years, attempts have been made to investigate indigenous drugs against infectious disease. *Trapa bispinosa* can be used as antimicrobial agent [20] which has evaluated antifungal activity of fruit extracts of different water chestnut varieties. A strong antifungal activity of ethanol and petroleum extract was found against the treated fungi resulting in remarkable inhibition zone in comparison to both dithane-Mfungicide and control. It was also evident that wild variety of water chestnut was comparatively more efficient in respect to antifungal activity compared to the red and green varieties of the same plant [5]. It was mentioned that the extracts of *Trapa bispinosa *showed interesting antimicrobial activity against Gram-positive and Gram-negative test organisms and significant cytotoxic activity [20].

### 10.10. Antibacterial Activity

Antibacterial activities of the fruit extract of two varieties (green and red) of water chestnut by the disc diffusion method from methanol extract were studied. The extract of red variety of water chestnut showed high antibacterial potential (31 mm) against *Bacillus subtilis* with the concentration of 600 micron. On the other hand, green variety showed highest antibacterial activities (12 mm) against both *Staphylococcus aureus* and *Shigellasonnei* with the concentration of 600 microgram Kanamycin used as standard. In this disc diffusion assay, the methanol extract of red variety was found to have a significant antibacterial efficiency compared to the extract of green variety of water chestnut. These findings pinpoint the efficiency of these extracts to inhibit microbial growth [20].

### 10.11. ABTS Scavenging Activity

ABTS scavenging assay is applicable for screening both lipophilic and hydrophilic antioxidants which shows the percentage inhibition of ABTS radical by *Trapa bispinosa *extracts and standard trolox. Acetone extract (IC50 = 5 ± 0.24 mg/mL) and DCM: Me OH (IC50 = 7 ± 0.76 mg/mL) showed less scavenging than that of standard trolox (IC50 = 1 ± 0.01 mg/mL). There was significant difference (*P* < 0.05) in ABTS scavenging activity of both the extracts (Gupta et al., 2012).

### 10.12. Enzymatic Activity

The activities of some enzyme like amylase, cellulose, invertase, lipase, and protease were studied in locally available two varieties (green and red) of water chestnuts, Asian aquatic fruits popular for its nutritive value and medicinal properties. All the tested enzyme activities were found slightly higher in green variety than in red variety. The amylase, cellulase, invertase, lipase, and protease activities were 0.3532, 0.1922, 0.0587, 0.0234, and 0.0548 mg/mL/min, respectively, in green variety and 0.2514, 0.1221, 0.0520, 0.0204, and 0.0515 mg/mL/min, respectively in red variety. From the enzyme activity assay it was found that water chestnuts might be used as a source of some enzymes such as amylase, cellulase, invertase, lipase, and protease. These enzyme activities could be major factor for determining the nutritive and medicinal value of the water chestnuts.

## 11. Pharmaceutical Uses

### 11.1. Excipients

Starch obtained from *Trapa bispinosa *has comparable physicochemical and binding activities compared to official starches. Physicochemical property of water chestnut starch (WCS) was comparatively evaluated with official potato and maize starch. The granule shape is round to oval with the particle size diameter 18–130 µm. The powder characteristics are nearby similar to the official starches. Hydration and swelling capacity of WCS is approximately similar which make this potential excipient in pharmaceutical formulation development. Thus, it has potential to be used as binder industrial [35].

### 11.2. Metal Chelation Activity

Lipid peroxidation by the Fenton reactions is initiated by ferrous iron. Thus, minimizing Fe^+2^ concentrations in Fenton reactions by metal chelation affords protection against oxidative damage. The chelating of Fe^+2^ ions by the extracts was estimated by the method of Dinis. In this assay, both extracts interfered with the formation of ferrous and ferrozine complex in an almost similar manner, suggesting that they have chelating activity and capture Fe^+2^ ion before ferrozine.

### 11.3. Freeze Thaw Stabilization

Among the four gums tested, GG was effective in increasing freeze thaw stability, when 0.2% gum was added; while at 0.3%, gum ACA was more effective than GG. It was noted that the addition of salts increases the stability of the gel towards low temperature. The addition of NaCl at 0.5%, 1%, and 2% showed maximum stability compared to other salts due to the hydrophilic nature of the sodium chloride enhanced water-holding ability of the starch pastes thereby limiting amount of water exuded but the reduced stability was observed in the presence of CaCl_2_. The addition of salts increased the stability of the mixtures against the freeze thawing at varying concentrations [27].

### 11.4. Starch as Additive in Pharmaceuticals

**Figure 4:**
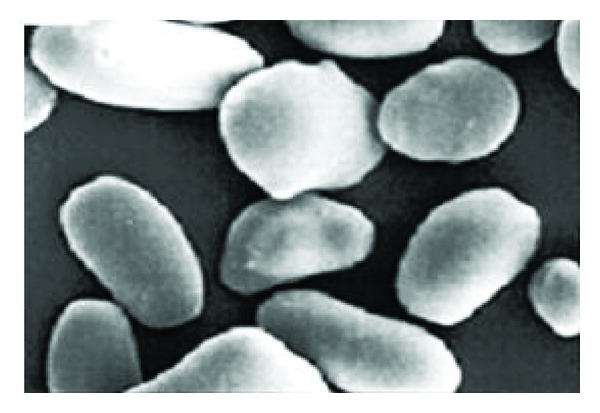
*Trapa bispinosa* starch grains.

The appearances of native *Trapa bispinosa* starches are shown in Figure 4. The surfaces of the granules of all samples are smooth with no evidence of cracks. Some granules appeared to be either round or oval in shape with “horn(s)” protruding from the surface [32]. The physicochemical properties of the starch extracted from krajub *Trapa bispinosa* were investigated. Scanning electron microscopy of the starch granules showed that they were either oval or round in shape with small horn(s) protruding from the surface. Amylose content of the krajub starch was 29.62% (dry weight basis dwb). The pasting temperatures of 6–8% starch suspension were 81–83°C. Bra bender amylogram showed no peak viscosity and very low breakdown, indicating high heat and shear stability of the starch suspension. The starch pastes highly retrograded and formed an opaque gel. The X-ray diffraction patterns of the starch revealed a C-type crystallite. The starch granules were more resistant to acid hydrolysis (2.2 NHCl at ambient temperature) than mung bean starch (C-type crystallite) [36].

## 12. Analytical Evaluation

### 12.1. Yoghurt Stabilizer

Enriched yoghurt with *Trapa bispinosa* starch at different levels was studied with physicochemical and sensory analysis. Yoghurt prepared by incorporation of *Trapa bispinosa* starch at concentration of 0.5%, 0.75%, 1%, and 1.25% was compared for these characteristics to the yoghurt containing stabilizer gelatin 0.5% w/w. Physiochemical parameters (fat, pH, acidity, synergies, water holding capacity, viscosity, protein, etc.) sensory evaluation, and microbial analysis (total viable count and coli form test) were studied. Use of *Trapa bispinosa *starch produced better results in terms of lowering synergies and increasing water holding capacity, viscosity, and overall acceptability for all sensory attributes. Addition of *Trapa bispinosa* starch did not influence the taste and overall acceptability. *Trapa bispinosa *starch 1.25% gave most excellent results for water holding capacity, synergies, and viscosity and *Trapa bispinosa *starch 0.75% gave most excellent results for all sensory attributes. Yoghurt shelf life was increased up to 25 days [26].

### 12.2. Molecular Identification

Reference [37] developed two marker systems for the molecular identification of three *Trapa* species based on the length variation of nuclear AP2 and trnL-F chloroplast intergenic spacer region and concluded that that nucleotide sequence variations can serve as a fast, reliable, and reproducible tool for molecular genotyping and examining the natural hybrid of water chestnut species.

### 12.3. Characterization and Antimicrobial Properties of Starch-Chitosan Edible Films

The characterization and antimicrobial properties of water chestnut starch-chitosan (WSC) films containing *Cornus* officinal are fruit extract (COE 1% w/w), glycerol monolaurate (GML 1% w/w), nisin (10,000 IU/g), and pine needle essential oil (PNEO 0.35% v/v), and their combinations were evaluated. Incorporation of COE decreased pH value of the film-forming solution, the moisture content, and the water absorption expansion ability (WAEA). GML-incorporated film had lower WAEA, tensile strength, elongation, and puncture strength. However, films with nisin displayed good mechanical properties. All the treated films were less transparent and higher in water vapour permeability values. For film microstructure, the presence of PNEO caused discontinuities with lipid droplets or holes embedded in a continuous network and the incorporation of GML led to abaisse-like structures. The COE, GML, nisin, PNEO and their combinations incorporated in the WSC films are effective in inhibiting the growth of *Escherichia coli* O157:H7, *Staphylococcus aureus*, and *Listeria monocytogenes* at different levels. The results showed that WSC films containing COE and GML, GML and nisin, and COE and nisin were able to reduce the number of *E. coli* O157:H7, *S. aureus*, and *L. monocytogenes*. This research has potential applications to the extension of the shelf life of food products [22].

### 12.4. Rheological Character of High-Amylose Starch

The molecular structure and rheological properties of high-amylose water caltrop (*Trapa bispinosa* Roxb) starch cultivated in Vietnam were investigated. The water caltrop starch had 47.1% amylose and its molecular weight (Mw) was (4.77 ± 0.27) × 10^6^ g/mol, whereas the Mw was (2.07 ± 0.10) × 10^7^ g/mol for amylopectin an extremely high storage modulus up to approximately 1,200 Pa. High-amylose water caltrop starch paste had an extremely high final viscosity compared to that of other cereal starches. These rheological behaviors may have been due to the extremely high amylose content [38].

### 12.5. Rapid Biosynthesis of Silver Nanoparticles

*Trapa bispinosa* has high reducing capacity to synthesize monodispersed silver nanoparticles (SNPs) within 120 seconds at 30°C which is the shortest tenure reported for SNP synthesis using plants. Moreover, we also instigated impact of different pH values on fabrication of SNPs using visible spectroscopy with respect to time. Percentage of conversion of Ag^+^ ions into Ag° was calculated using ICP-AES analysis and was found to be 97% at pH = 7. To investigate the reduction of Ag^+^ ions to SNPs, cyclic voltammetry (CV) and open circuit potential (OCP) using 0.1 MKNO_3_ were performed. There was prompt reduction in cathodic and anodic currents after addition of the peel extract which indicates the reducing power of *T. bispinosa* peel. Stability of the SNPs was studied using flocculation parameter (FP) which was found to be the least at all the pH values. FP was found to be indirectly proportional to stability of the nanoparticles [25].

## 13. Conclusion

The systematic review of Unani, Ayurvedic literature indicates that *Trapa bispinosa* has immense potential in the treatment of conditions such as diarrhoea, strangury, dysuria, polyuria, sexual debility, general debility, sore throat, and lumbago. The recent pharmacological studies reveal that this has important analgesic, antibiotic, antidiabetic and immunomodulatory activities. The global interest toward traditional medicines is increasing due to the safe and time tested remedies with lesser side effects. This review directs *Trapa bispinosa* as a potentially safe and effective plant that has immense medicinal and nutritional values and benefits.

## Figures and Tables

**Figure 2:**
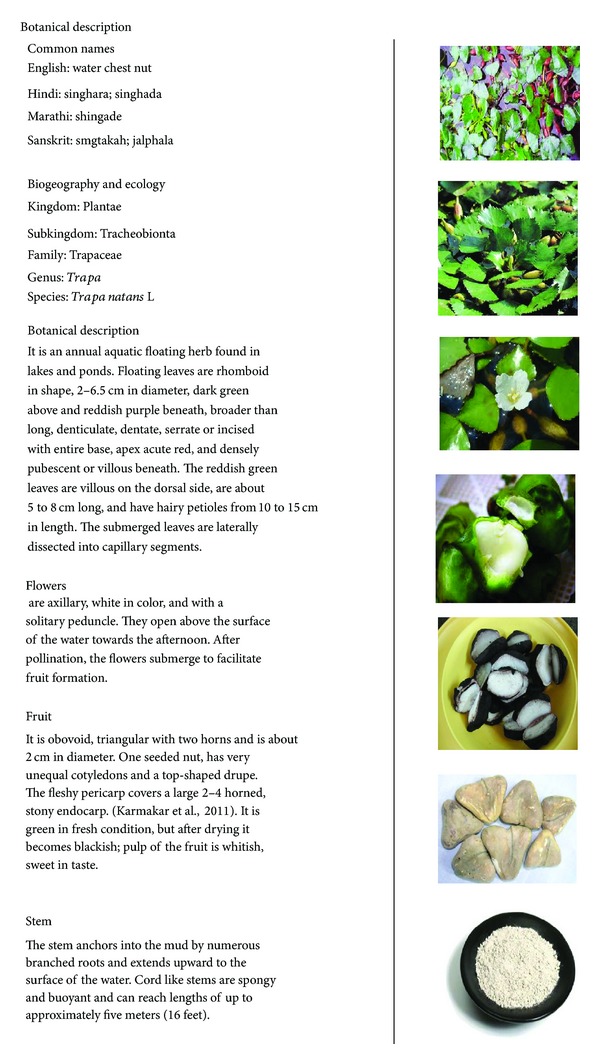
Botanical description of plant *Trapa bispinosa* Roxb [32].

**Figure 3:**
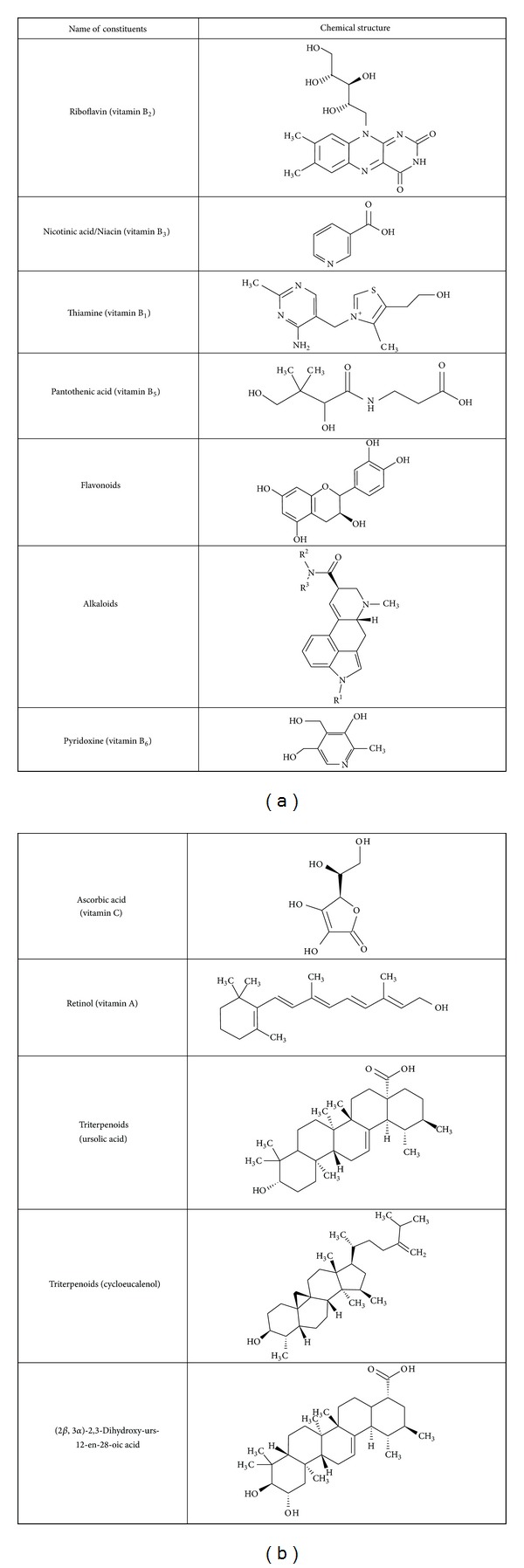
Chemical constituents present in the plant of *Trapa bispinosa*.

**Table 1:** Physicochemical Characteristics of *Trapa bispinosa. *

Constituent	Percentage (wet basis)
Moisture	81.12 ± 0.5
Total soluble solids (°Brix)	7.2 ± 0.2
Total acidity	0.142 ± 0.03
Crude lipids	0.36 ± 0.02
Total ash	1.33 ± 0.04
Crude fiber	0.72 ± 0.02
Total proteins	1.87 ± 0.03

**Table 2:** Mineral composition of *Trapa bispinosa *on concentration basis (ppm).

Minerals	Content in *Trapa bispinosa *
Ca	365 ± 0.23
K	98.2 ± 1.23
Na	37.24 ± 0.36
Zn	6.926 ± 0.12
Ba	0.482 ± 0.32
Cr	0.106 ± 0.02

**Table 3:** Total Phenolic and total flavonoids content of extracts of *Trapa bispinosa*.

Extract	Total phenolic content (µg GAE/mg extract)	Total flavonoids content (µg GAE/mg extract)
Acetone DCM : MeOH	7.924 ± 0.03 : 743.38 ± 0.35	3.924 ± 0.01 : 491.37 ± 0.56

**Table 4:** A summary of reported pharmacological activity of *Trapa bispinosa *Roxb.

Species/method used	Property	Source
Rats	Immunomodulator	[10]
	Nootropic, neuroprotective	[15]
	Antiulcer	[16]
	Analgesic	[17]
	Antidiabetic	[18]

Female Swiss albino mice	Neuroprotective	[19]

Fungi	Antifungal	[20]
	Antifungal peptides	[21]
	Antimicrobial and enzymatic activity	[22]
	Antimicrobial activity and cytotoxicity	[14]

On starch-chitosan edible films	Antimicrobial properties	[22]

*Staphylococcus aureus*, *Bacillus subtilis*, *Bacillus megaterium*, *Sarcinallutea*, and *Bacillus cereus *	Antibacterial Activity	[20]

Yeast leavened breads	Physical and sensory properties of starch	[23]

	Starch as pharmaceutical, binder in solid dosage form	[4]
	Nutritional, photochemical and antioxidant	[24]
	Characteristics of starch	[25]
	Stabilizer	[26]
	Starch as excipient in tablet manufacturing	[27]
	Yogurt as a stabilizer	[26]

In vitro methods	Antioxidantactivity	[8]
	Anticancer activity	[28]
	Enzymatic activity	[22]
